# Links of gut microbiota composition with alcohol dependence syndrome and alcoholic liver disease

**DOI:** 10.1186/s40168-017-0359-2

**Published:** 2017-10-17

**Authors:** Veronika B. Dubinkina, Alexander V. Tyakht, Vera Y. Odintsova, Konstantin S. Yarygin, Boris A. Kovarsky, Alexander V. Pavlenko, Dmitry S. Ischenko, Anna S. Popenko, Dmitry G. Alexeev, Anastasiya Y. Taraskina, Regina F. Nasyrova, Evgeny M. Krupitsky, Nino V. Shalikiani, Igor G. Bakulin, Petr L. Shcherbakov, Lyubov O. Skorodumova, Andrei K. Larin, Elena S. Kostryukova, Rustam A. Abdulkhakov, Sayar R. Abdulkhakov, Sergey Y. Malanin, Ruzilya K. Ismagilova, Tatiana V. Grigoryeva, Elena N. Ilina, Vadim M. Govorun

**Affiliations:** 1 Moscow Institute of Physics and Technology, Institutskiy per. 9, Dolgoprudny, Moscow Region, 141700 Russia; 2 Federal Research and Clinical Center of Physical-Chemical Medicine, Malaya Pirogovskaya 1a, Moscow, 119435 Russia; 3 Department of Bioengineering, University of Illinois at Urbana-Champaign, 1304 W. Springfield Avenue Urbana, Champaign, IL 61801 USA; 4 Carl R. Woese Institute for Genomic Biology, 1206 West Gregory Drive, Urbana, IL 61801 USA; 5 ITMO University, Kronverkskiy pr. 49, Saint-Petersburg, 197101 Russia; 6 Saint-Petersburg Bekhterev Psychoneurological Research Institute, Bekhtereva 3, Saint-Petersburg, 192019 Russia; 7 Moscow Clinical Scientific Center, Shosse Entuziastov 86, Moscow, 111123 Russia; 8 Kazan State Medical University, Butlerova 49, Kazan, 420012 Russia; 9 Kazan Federal University, Kremlyovskaya 18, Kazan, 420008 Russia

**Keywords:** Metagenome, Human gut microbiota, Alcoholic dependence syndrome, Alcoholic liver cirrhosis, *Bifidobacterium*, *Lactobacillus*, Gut-brain axis, Virulence factors, Acetaldehyde

## Abstract

**Background:**

Alcohol abuse has deleterious effects on human health by disrupting the functions of many organs and systems. Gut microbiota has been implicated in the pathogenesis of alcohol-related liver diseases, with its composition manifesting expressed dysbiosis in patients suffering from alcoholic dependence. Due to its inherent plasticity, gut microbiota is an important target for prevention and treatment of these diseases. Identification of the impact of alcohol abuse with associated psychiatric symptoms on the gut community structure is confounded by the liver dysfunction. In order to differentiate the effects of these two factors, we conducted a comparative “shotgun” metagenomic survey of 99 patients with the alcohol dependence syndrome represented by two cohorts—with and without liver cirrhosis. The taxonomic and functional composition of the gut microbiota was subjected to a multifactor analysis including comparison with the external control group.

**Results:**

Alcoholic dependence and liver cirrhosis were associated with profound shifts in gut community structures and metabolic potential across the patients. The specific effects on species-level community composition were remarkably different between cohorts with and without liver cirrhosis. In both cases, the commensal microbiota was found to be depleted. Alcoholic dependence was inversely associated with the levels of butyrate-producing species from the *Clostridiales* order, while the cirrhosis—with multiple members of the *Bacteroidales* order. The opportunist pathogens linked to alcoholic dependence included pro-inflammatory *Enterobacteriaceae*, while the hallmarks of cirrhosis included an increase of oral microbes in the gut and more frequent occurrence of abnormal community structures. Interestingly, each of the two factors was associated with the expressed enrichment in many *Bifidobacterium* and *Lactobacillus*—but the exact set of the species was different between alcoholic dependence and liver cirrhosis. At the level of functional potential, the patients showed different patterns of increase in functions related to alcohol metabolism and virulence factors, as well as pathways related to inflammation.

**Conclusions:**

Multiple shifts in the community structure and metabolic potential suggest strong negative influence of alcohol dependence and associated liver dysfunction on gut microbiota. The identified differences in patterns of impact between these two factors are important for planning of personalized treatment and prevention of these pathologies via microbiota modulation. Particularly, the expansion of *Bifidobacterium* and *Lactobacillus* suggests that probiotic interventions for patients with alcohol-related disorders using representatives of the same taxa should be considered with caution. Taxonomic and functional analysis shows an increased propensity of the gut microbiota to synthesis of the toxic acetaldehyde, suggesting higher risk of colorectal cancer and other pathologies in alcoholics.

**Electronic supplementary material:**

The online version of this article (10.1186/s40168-017-0359-2) contains supplementary material, which is available to authorized users.

## Background

The majority of adult population consumes alcohol in various quantities, and alcoholism is the leading cause of premature deaths in the world [1]. Long-term alcohol abuse exerts a spectrum of potent effects on different body systems, ranging from psychiatric symptoms, malnutrition and chronic pancreatitis to alcoholic liver disease, hepatocellular carcinoma, and coronary heart disease. Alcoholism increases the risk of oropharyngeal and esophageal cancer. Central and peripheral nervous systems are vulnerable and strongly affected by ethanol. As diet is one of the major factors affecting the community structure and functional potential of gut microbiota [2], alcohol and the products of its degradation by human organism can strongly modulate the human gut microbiota [3]. Moreover, gut dysbiosis contribute to neuroinflammation in the context of alcohol exposure and withdrawal, subsequently leading to the psychiatric symptoms of alcoholism—an understanding concordant with the concept of “gut-brain axis” [4]. However, most studies of alcohol dependence syndrome (ADS) are focused on examining the neurophysiologic effects as well as its action on the functions of liver and other organs, while the question of how alcohol dependence impacts the gut microbiota to a large extent remains unexplored. Recently, the application of cultivation-free approaches (qPCR and 16S rRNA metagenomic sequencing) demonstrated that the extent of alcohol impact on gut microbiota varies between individuals [5]. In a group of patients with ADS, it was shown to be associated with a decreased abundance of major commensal microbial taxa, including *Roseburia*, *Faecalibacterium*, *Blautia*, *Bacteroides*, and *Lachnospiraceae*, increased intestinal permeability, and inflammation [6–8].

Alcoholism is commonly accompanied by a number of pathologies each of which can individually shift the composition of the gut microbiota. Particularly, chronic alcohol abuse is a factor strongly contributing to the pathogenesis of various liver diseases [9]. Alcoholic liver cirrhosis (ALC) develops in 10–15% of alcoholics [10]. A growing number of recent studies support the hypothesis that the gut microbiota can play a significant role in the onset and progression of the alcoholic liver disease (ALD) [11, 12], nonalcoholic fat liver disease (NAFLD) [13], nonalcoholic steatohepatitis (NASH) [11], and other liver dysfunctions. One of the proposed mechanisms is via a direct impact of the microbial products like endotoxins on liver, as it interacts with the gut both directly via the hepatic artery as well as indirectly via the metabolism of bile acids [14]. However, the mechanisms of such interactions are yet to be elucidated. The gut microbial community of patients with ALC is known to be dysbiotic; its composition is characteristic by increased presence of *Proteobacteria* (particularly, *Gammaproteobacteria)* and *Bacilli*. These shifts are mirrored by a decrease in the commensal taxa *Clostridia*, *Bacteroidetes*, and *Ruminococcaceae*, as well as *Lactobacillus* and *Bifidobacterium*. A significant reduction of *Lachnospiraceae*, *Roseburia*, *Faecalibacterium*, and *Blautia* levels contributes to intestinal bacterial overgrowth, which, together with the acetaldehyde-caused increased intestinal permeability, leads to an increased blood concentrations of endotoxins and activation of inflammatory cascades likely inducing liver damage [7, 15–17].

Liver dysfunction is a major confounding factor in surveys of gut microbiota in patients with ADS. In order to identify the changes on microbiota composition and functions resulting from alcohol dependence and liver disease for the first time we conducted a “shotgun” metagenomic analysis of stool samples from a group of patients suffering from alcoholism represented by two cohorts—patients with ADS (without advanced liver disease) and patients with ALC (with advanced liver disease, i.e., liver cirrhosis). In order to disentangle the effects of these two pathologies, we carried out a multifactor statistical analysis of the metagenomic data. Moreover, we analyzed changes in the specific functional potential of microbiota linked to both virulence factors and alcohol metabolism.

## Results

### Cohorts of patients

In total, 99 patients were enrolled in the study carried out at three clinical centers in three Russian cities (see Table 1, the “Methods” section, and Additional file 1: Table S1). The cohort included 72 patients with ADS, and 27—with ALC (Additional file 2: Table S2); for a third of the cohort, the gut metagenome analysis has been performed and its results have been previously published [18, 19]; experimental data were made publicly available [20]. Sequencing of the stool samples yielded 25.8 ± 16.1 M of 50 bp reads per sample (127.5 Gbp in total).

**Table 1:** Summary information about the patients and the control group. The values are mean ± s.d., here and below

	*N*	Age	Gender	BMI
Patients with ADS	72	44 ± 10	3 F/69 M	23.3 ± 3.9
Patients with ALC	27	49 ± 7	5 F/22 M	27.4 ± 4.1
External control group [58]	60	36 ± 11	32 F/28 M	25.7 ± 5.6

### Gut microbial communities of the patients with ADS and ALC differ from each other and have disease-specific compositions

**Fig. 1:**
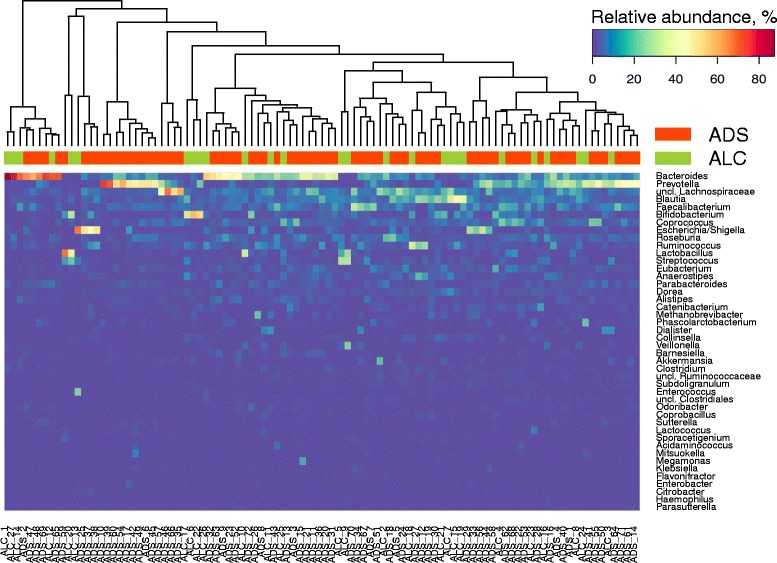
The most prevalent genera in the gut microbiota of patients with ADS and ALC. The columns correspond to the samples/patients; the patient group is denoted with a top color bar. The figure shows the taxa with the relative abundance of ≥ 1% in at least one of the metagenomes. Each row name starting with “uncl.” corresponds to total relative abundance of all unclassified genera belonging to the respective taxon of higher order (e.g., family or order). The hierarchical clustering was performed using the Euclidean metric and complete linkage

Mapping of metagenomic reads to the reference catalogue of gut microbial genomes (see the “Methods” section) allowed us to identify 246 microbial species (determined as the number of detected genomes) belonging to 79 genera in our cohort of ADS patients and 230 species from 71 genera in our cohort of ALC patients (see Fig. 1; the complete composition is in the Additional files 3 and 4: Table S3, S4). MetaPhlAn2 method based on unique clade-specific markers [21] generated similar taxonomic composition represented by 334 and 281 species for ADS and ALC cohorts, respectively (Additional files 5 and 6: Table S5, S6). The richness of the gut community (alpha-diversity) in either ADS or ALC patients was not statistically different from the control group (Shannon index 3.3 ± 0.6 vs. 3.3 ± 0.6 and 3.2 ± 0.5, *p* = 0.8 and 0.2, respectively, Welch’s test).

The analysis of the relative abundance of the species (Additional file 7: Figure S1) demonstrated that the gut community in patients is generally dominated by commensal species typical for the healthy populations of the world [22]. The three most represented genera for the ADS patients included *Bacteroides* (relative abundance 19.2% ± 18.2%), *Prevotella* (16.3% ± 20.1%), and *Faecalibacterium* (6.7% ± 5.7%). For the ALC group, the respective list included *Bacteroides* (19.6% ± 27.7%), *Blautia* (9.7% ± 10.9%), and *Bifidobacterium* (9.4% ± 15.9%).

A direct comparison of the gut community structures between each of the two groups of our patients and the control group allowed us to identify a number of taxa that were differentially abundant (Additional files 8 and 9: Tables S7, S8). Particularly, the ADS patients had higher levels of two genera (*Klebsiella*, *Lactococcus*) and four species (*K. pneumoniae*, *Lactobacillus salivarius*, *Citrobacter koseri*, *Lactococcus lactis* subsp. *cremoris*) than the control group. For the ALC patients, the respective list included two genera and eight species: *Bifidobacterium* (*B. longum*, *dentium*, and *breve*) and *Streptococcus* (*S. thermophilus* and *mutans*), as well as multiple *Lactobacillus* species (*L. salivarius*, *antri*, and *crispatus*) (Mann-Whitney test, FDR-adjusted *p* value < 0.01). On the other hand, the taxa decreased in comparison with the healthy population included for ADS patients—3 genera (*Akkermansia*, *Coprococcus,* unclassified *Clostridiales*) and 19 species; for ALC patients—49 species from 13 genera (particularly, *Prevotella*, *Paraprevotella*, and *Alistipes*). The results were mainly in agreement with a similar analysis performed using MetaPhlAn2 (Additional files 10 and 11: Tables S9, S10).

Besides the microbial components, the MetaPhlAn2 allowed us to identify viral components of the gut metagenomes of our patients. Viruses were identified only in a few of our samples and in low abundance. Particularly, the most prevalent viruses were unclassified C2-like (max. 3.7% of the total relative abundance, in *n* = 21 patients), Dasheen mosaic (max. 0.07%, *n* = 22), and wheat dwarf viruses (max. 0.4%, *n* = 10). The list of detected bacteriophages included 10 viruses with hosts belonging to *Lactobacillus*, *Leuconostoc*, *Lactococcus*, *Streptococcus*, *Enterobacteria*, and other taxa. However, a comparison with the control group did not find any viruses that were significantly under- or overabundant in the guts of the ADS or ALC patients relative to the control group.

**Fig. 2:**
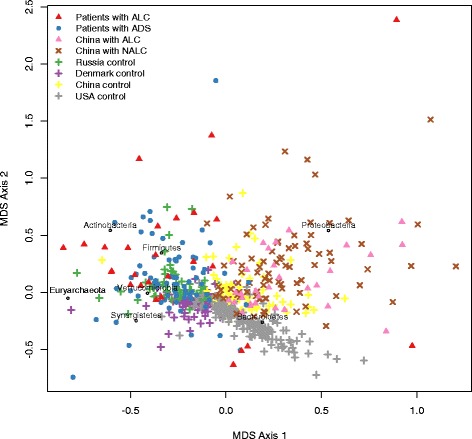
Distribution of the gut community structures of patients with ADS and ALC among world’s population. Multidimensional scaling (MDS) biplot using the Bray-Curtis dissimilarity metric. The labels denote the directions of increasing abundance in respective microbial phyla (only the phyla detected in > 4 metagenomes are shown)

The visualization of the distribution of the taxonomic composition in ADS and ALC patients in the context of the published data on world populations (Fig. 2) showed that ALC patients tend to be more shifted from the Russian control group than the ADS patients.

Among samples forming “outliers” on the MDS plot were two samples from the ADS group (ADS_59, ADS_39) and four samples from the ALC group (ALC_13, ALC_9, ALC_20, ALC_1). Species-level analysis showed that three of these samples were outlying due to the absolute dominance of a single commensal microbial genus and had associated low community richness (alpha-diversity): ADS_39 (dominated by *Prevotella* genus, 71%; Shannon index 1.65), ALC_1 (*Bacteroides*, 76%; Shannon index 2.34) and ADS_59 (64% *Lactobacillus*; Shannon index 1.79).

The remaining outliers were characterized by abnormal community structures dominated by a set of several bacterial species, most of which are known to be associated with gut inflammation. The sample ALC_13 included 68% of *Escherichia* spp., 22% of *Enterococcus*, and 7% of *Streptococcus*. Samples ALC_9 and ALC_20 had increased proportions of *Streptococcus salivarius* and *vestibularis* (totally 27 and 18%, respectively), as well as *Lactobacillus salivarius* and *crispatus* (20 and 36%). The *Veillonella* genus known to be prevalent in buccal microbiota was another genus abnormally overrepresented in the ALC_9 sample (*V. atypica*, *parvula*, and *dispar* totally accounting for 17% of the microbial abundance). Intriguingly, the ALC_20 sample had a high fraction of *Bifidobacterium* species known by its probiotic representatives (30%, *B. longum* subsp. *longum* and subsp. *infantis*).

As an alternative method for detecting the metagenomes with uncommon composition, we applied the reference-free MetaFast algorithm based on the adaptive de novo assembly of combined metagenomic reads [23]. The outliers detected using this algorithm (Additional file 12: Figure S2) contained only the outliers previously detected using the reference mapping. This indicates that no gut communities were strongly dominated by unknown components (e.g., viruses or fungi) or significantly affected by technical artifacts in both groups of patients.

### Prevalence of buccal species is specific to liver cirrhosis but not alcohol dependence

A recent metagenomic survey of the gut microbiota in patients with liver cirrhosis has shown a significant enrichment of bacteria normally inhabiting oral cavity [16] which is likely due to an impaired liver function. We assessed the presence of such species in the gut microbiota of the patients with ADS and ALC (Table 2).

**Table 2:** Enrichment of the potentially buccal microbial species in gut microbiota of the patients. The ordered list includes the species with > 1% abundance in at least one sample; the detections of the ADS patients are filled with gray

Sample ID	Microbial species	Relative abundance, %
ALC_9	*Lactobacillus salivarius*	28.67
ALC_20	*Lactobacillus salivarius*	25.84
ALC_5	*Lactobacillus salivarius*	13.55
ALC_10	*Lactobacillus salivarius*	13.14
ALC_9	*Streptococcus salivarius*	11.56
ALC_5	*Streptococcus salivarius*	11.2
ALC_25	*Lactobacillus salivarius*	8.13
ADS_1	*Lactobacillus salivarius*	7.69
ADS_67	*Streptococcus salivarius*	5.22
ALC_9	*Veillonella atypica*	4.55
ALC_5	*Streptococcus parasanguinis*	4.37
ALC_3	*Streptococcus vestibularis*	4.04
ALC_9	*Streptococcus parasanguinis*	3.83
ADS_30	*Lactobacillus salivarius*	3.38
ALC_20	*Streptococcus salivarius*	3.16
ADS_34	*Veillonella parvula*	3.14
ALC_3	*Streptococcus salivarius*	3.01
ALC_16	*Lactobacillus salivarius*	2.96
ADS_8	*Veillonella parvula*	2.88
ADS_12	*Streptococcus salivarius*	2.79
ADS_59	*Lactobacillus salivarius*	2.65
ALC_10	*Veillonella parvula*	2.64
ADS_28	*Streptococcus salivarius*	2.33
ALC_3	*Streptococcus parasanguinis*	2.3
ALC_20	*Streptococcus vestibularis*	2.16
ADS_28	*Lactobacillus salivarius*	2.09

The highest fractions of potentially oral species tend to occur in ALC patients rather than in ADS patients (for the latter, the levels are quite low). The complete comparison of ranks (see the “Methods” section) for each species showed that *Streptococcus constellatus*, *Streptococcus salivarius*, *Veillonella atypica*, *Veillonella dispar*, and *Veillonella parvula* have significantly higher ranks in the microbiota of ALC than of ADS patients (one-tailed Mann-Whitney test, *p* < 0.05). It suggests that alcoholic dependence itself is linked to an enrichment of oral species in the gut microbiota to a lesser degree than the alcohol liver cirrhosis.

### Effect of alcohol dependence and liver cirrhosis on gut community structure is different

**Fig. 3:**
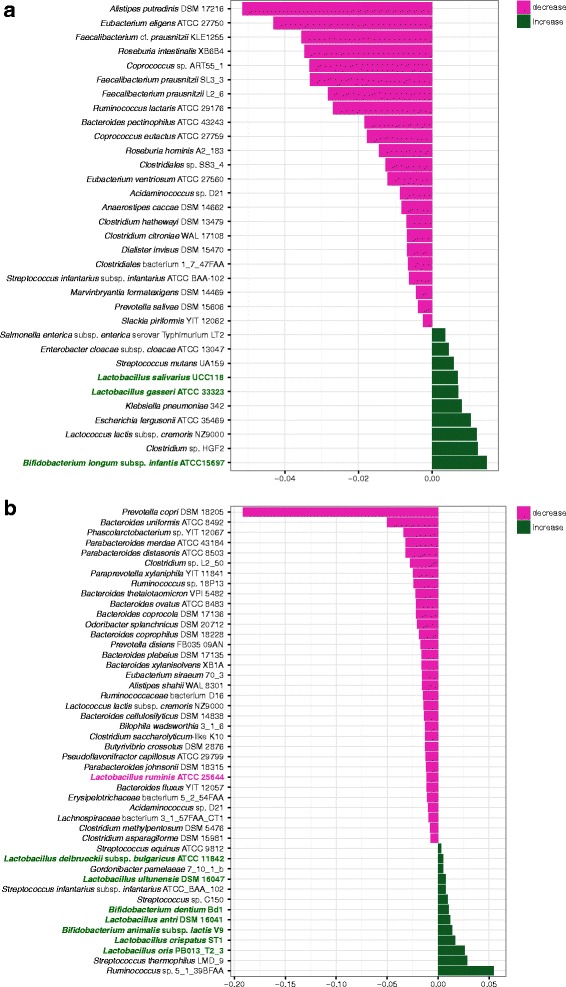
Microbial species significantly associated with alcohol dependence and liver disease. The figure shows coefficients of the linear model obtained by applying MaAsLin method to reference-mapping based taxonomic composition vectors (adjusted *p* value < 0.05). Positive values denote a direct association between the clinical factor and the relative abundance of the respective taxon, while negative values denote a reverse association. *Bifidobacterium* and *Lactobacillus* species are highlighted according to the direction of the respective association. **a** Alcohol dependence. **b** Liver cirrhosis

In order to identify the influence of each of the clinical factors on taxonomic composition of the gut microbiota, we performed a multifactor analysis using MaAsLin package [24]. Metagenomes from the Russian population—both our patients as well as healthy subjects—were included. Analysis of the variance using PERMANOVA (Bray-Curtis dissimilarity matrix) revealed three factors significantly linked to the microbiota composition: liver cirrhosis (explained variance *R* ^2^ = 3.2%, adj. *p* = 0.002), alcohol dependence (*R* ^2^ = 2.9%, adj. *p* = 0.002), and gender (*R* ^2^ = 1.6%, adj. *p* = 0.007); association with age was not significant (adj. *p* = 0.129) (Bray-Curtis dissimilarity index, 1000 permutations). After correction for the effect of the gender, both liver cirrhosis and alcohol dependence were found to still have a large impact on gut microbiota (Fig. 3; complete results are listed in Additional files 13 and 14: Table S11, S12).

Liver cirrhosis was shown to be associated with changes in relative abundances of 8 genera and 46 species. Taxa with decreased levels include *Parabacteroides* genus (including its members *P. distasonis*, *johnsonii*, and *merdae*), *Prevotella* (*P. copri* and *disiens*), *Clostridium* (*C. asparagiforme*, *methylpentosum*, *saccharolyticum*-like K10, and sp. L2–50), *Paraprevotella xylaniphila*, *Odoribacter splanchnicus*, *Phascolarctobacterium* sp. YIT 11841, and nine species from the *Bacteroides* genus as well as other commensal gut microbes. On the other hand, an increase was observed for the *Gordonibacter pamelaeae*, *Ruminococcus* sp. 5_1_39BFAA and, interestingly, for multiple members of the genera *Lactobacillus* and *Bifidobacterium*: *L. antri*, *crispatus*, *delbrueckii*, *oris*, *ultunensis*, and *B. animalis* and *dentium*.

A metagenomic signature of alcohol dependence, the second strong factor affecting the gut microbiota, was found to include 6 genera and 34 species that only slightly overlapped with the signature of liver cirrhosis. Changes uniquely associated with alcoholism include an increased abundance of the *Klebsiella* genus and decreased abundances of *Coprococcus*, *Faecalibacterium prausnitzii*, and unclassified *Clostridiales*. At the same time, certain changes in the gut microbiota were collinear with those detected in the signature of liver cirrhosis: both pathologies were marked by a decrease of *Acidaminococcus* sp. D21. As in the case of the cirrhosis, alcohol dependence was associated with an increased fraction of several *Lactobacillus* and *Bifidobacterium* members, but due to different species—*B. longum* and *L. gasseri* and *salivarius*.

### Functions of microbiota in alcoholics manifest increased propensity toward alcohol metabolism and enrichment in virulence factors

Shotgun metagenomics allowed us to assess the total functional potential of the gut microbiota and compare it between healthy subjects and each of the groups of alcoholic patients. We identified major changes in the metabolic potential at the level of metabolic pathways. For the ALC patients, seven KEGG pathways were significantly increased and three—decreased in comparison with the control group (Additional file 15: Table S13). Metabolic potential of microbiota in ADS patients was also affected but mainly characterized by a decrease of certain functions: only the phosphotransferase system (PTS) pathway was increased while eight pathways were found to be decreased in ADS relative to healthy controls (Additional file 16: Table S14).

In order to explore the functional shift in patients’ microbiota on a more detailed level, we examined three specific functions performed by the gut microbiota that might be interlinked with severity of alcoholic dependence and liver cirrhosis. The first one is the ability of the gut microbial community to transform ethanol and its metabolites including toxic ones like acetaldehyde that can induce colorectal cancer [25]. The second function is biotransformation of bile acids: microbiota is an indispensable in the chain of conversion of bile acids in a human organism [26]. Furthermore, liver damage arising as a consequence of alcohol abuse is commonly linked to dysregulation of bile volume and composition. Thirdly, increased gut permeability observed in alcoholics might lead to passage of microbes and their metabolites through the intestinal wall and result in increased plasma endotoxin levels, therefore playing a crucial role in developing cirrhosis and its complications (e.g., spontaneous bacterial peritonitis) [27]. Therefore, virulence factors were selected as the third group for the functional analysis. We evaluated changes in the gut microbiota potential to perform these three functions by comparing relative abundances of the respective genes between healthy subjects and each of our ADS and ALC cohorts.

**Fig. 4:**
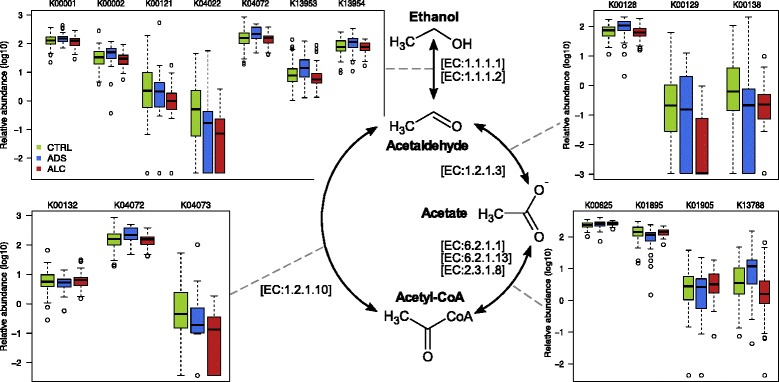
Potential of metabolic reactions related to alcohol metabolism in gut metagenomes. Boxplots show distribution of KEGG Orthology Groups relative abundance for ALC and ADS patients and control group

The gut microbiota in both ADS and ALC cohorts was significantly enriched in functions related to alcohol metabolism: out of 19 analyzed gene groups, 6 and 9 had increased relative abundance in ADS and ALC cohorts, respectively (Mann-Whitney test, adj. *p* < 0.05; see Fig. 4 and Additional file 17: Table S15). Most of the genes belonged to the family of alcohol, aldehyde, and acetaldehyde dehydrogenases. An inverse trend—decrease in ADS and ALC cohorts in comparison with the control group—was observed only for 3 and 2 KO groups belonging to other gene classes.

Interestingly, we did not detect significant changes for any of the bile metabolism genes neither for the ALC nor for the ADS group. On the other hand, functions related to the virulence appeared to be strongly increased in metagenomes of ADS patients: 50 virulence factor genes were significantly increased in comparison with healthy subjects (Additional file 18: Table S16). According to the taxonomic annotation from the VFDB database, they belong to microbes from *Enterobacteriaceae* family—this fact is in agreement with previously mentioned increased prevalence of the *Escherichia* and other members of the family in the microbiota of ADS patients. On the other hand, metagenomes of ALC patients compared to healthy controls did not manifest any differences in functions related to virulence factors.

## Discussion

Several metagenomic studies have outlined the gut microbiota composition of patients with ALC [16, 28] and ADS [5, 29]—however, all of the projects describing microbiota in ADS were based on 16S rRNA amplicon sequencing. To the best of authors’ knowledge, the present study is the first one to describe the gut metagenome of ADS patients using shotgun (whole-genome) metagenomics and compare both its taxonomic as well as functional composition with ALC patients and healthy population. Our study of two groups of alcoholics with different degree of liver dysfunction demonstrated that the most expressed feature of each group is a substantial reduction of many commensal gut taxa, while the number of increased species is rather low. Such relative under representation of the “positive” markers in the pathology resembles a “burst of microbiota”—when shifts away from the balanced community structures have different directions that strongly vary between subjects. We found that patients with ALC manifest these “bursts” even to a greater extent than ADS patients—due to the combined impact of alcohol dependence and liver dysfunction. Visualization highlights extreme cases of this phenomenon as outliers. We confirmed the existence of such outlying samples using an alternative reference-free analysis of shotgun metagenomes.

Notably, the “drivers” of dysbiotic communities in ADS patients and ALC patients were found to be different. In an attempt to improve the precision of the taxonomic comparison, based on one common feature of our two groups of patients (i.e., alcohol dependence), we conducted a multifactor analysis using a state-of-art algorithm based on boosted general linear models. The results suggest that each of two factors, liver dysfunction and alcohol dependence, is associated with a significantly changed abundance of distinct bacterial taxa representing potential biomarkers of these two diseases. Interestingly, metagenomic signatures of two pathologies contain nonoverlapping set of markers; and few of the shared markers manifest opposite directions of change.

Liver cirrhosis is characterized by a higher level of gut dysbiosis than alcoholism. Massive depletion of major commensals from the *Bacteroidales* order is accompanied by a rise of taxa normally inhabiting the oral cavity, as identified during our analysis of relative abundances of respective genomes. This effect is likely linked to the abnormal bile secretion and composition and is in agreement with the results of an earlier study describing gut metagenome in nonalcoholic liver cirrhosis patients [16], thus confirming this hallmark of the liver dysfunction on another cohort. The presence of oral species *Lactobacillus salivarius*, *Veillonella parvula*, and *Streptococcus salivarius* is more pronounced in ALC patients in comparison with both healthy controls as well as the ADS group. Interestingly, we found that *Bifidobacterium* abundance was significantly associated with the cirrhosis. This observation might appear somewhat contradictory to a well-known enrichment of species exhibiting probiotic properties within this genus. While one cannot completely rule out the possibility that some of these taxa, along with the *Streptococcus* and *Lactobacillus*, could be transient food-associated microbes, there is a growing body of evidence suggesting that levels of *Bifidobacterium* can increase in pathologies like alcoholic hepatitis [30] and inflammatory bowel diseases [31]*.* Overall, it would be interesting to compare the shifts in microbiota composition with detailed data on liver function assessed using additional methods like liver biopsy (although the latter was not performed in our study as it is not a required standard part of examination of such patients and possesses certain risks).

Alcohol dependence syndrome was found to be the second most important factor contributing to changes in microbiota. Inter-individual variability of types and amount of consumed drinks could have affected the results—however, due to low reliability of self-reported data on alcohol consumption, this was not in the focus of the study. Considering the metagenomic signature, the range of decreased autochthonous taxa in patients suffering from alcoholic dependence was found to be quite different from the ones of liver cirrhosis and includes many butyrate-producing taxa from the *Clostridiales* order, among them—*Faecalibacterium prausnitzii*, *Coprococcus eutactus*, and *Roseburia* spp. Depletion of the gut community potential for producing butyrate, a vital “currency” metabolite providing anti-inflammatory protection and oxidative stress amelioration in the gut, is coupled with the uprise of opportunist species tolerant to the reactive oxygen species (ROS), beneficiaries of the inflammation: *Enterococcus* and members of *Enterobacteriaceae* family (including *Escherichia* and *Klebsiella*). The intestinal domination of each of these opportunist genera was previously found to be associated with a high risk of bacteremia [32]. Due to an increased intestinal permeability observed in alcoholics, the dominance of these taxa is likely to lead to an increased passage of microbial products into *lamina propria* and trigger a stronger immune response than in a healthy population with similar community structures. Noteworthy, a strong association of alcoholism with *Clostridium* sp. HGF2, a species able to use carbon monoxide as an electron donor, apparently reflects the selective advantage conveyed by this ability in inflammatory conditions [33].

We found that both alcohol dependence and liver cirrhosis have certain commonalities in their metagenomic signatures. The central point is an increase of multiple species belonging to *Lactobacillus* and *Bifidobacterium* genera (although the precise set of differentially abundant species varies between these two pathologies). ALC patients tend to have a larger number of *Lactobacillus* species increased in abundance (among them oral species), while ADS patients are uniquely characterized by a statistically significant increase of the whole genus. We suggest that this could be associated with genus’ ability to metabolize alcohol and its products [34]. Noteworthy, the detected increase of *Bifidobacterium* is contrary to previous observations for the Japanese cohort of alcoholics. We speculate that two important factors could play role in contributing to these differences. The first factor is genetic difference between the cohorts from the two studies. Compared to the population of Russia, the East Asian people often carry genetic variants of alcohol metabolism genes that are associated with increased acetaldehyde levels after ethanol consumption [35]. Besides worse physiological responses to alcohol, this specific trait in the dynamics of ethanol degradation by the host is likely to modulate the gut microbiota community structure in the Japanese.

The second suggested factor is hazardous drinking with consumption of manufactured ethanol-based liquids not intended for consumption specific for Russia. Nonbeverage alcohol is a potentially major contributor to the mortality [36]. Its consumption results in higher frequency of episodes of very high blood concentrations of ethanol and its metabolites—comparing to heavy drinkers who consume equal amounts of ethanol in the form of beverage alcohol. Moreover, such surrogate products contain toxic aliphatic alcohols [37]. Apparently, these distinctive features also affect the way gut microbiota is changed in ADS patients—possibly, leading to increased fraction of *Bifidobacterium* in ADS patients from Russia. Members of the genus are potentially important acetaldehyde accumulators—species able to accumulate acetaldehyde in the colon in mutagenic concentrations [25]. Therefore, the observed enrichment of this genus coupled with a significantly increased abundance levels of microbial ADH and other genes related to alcohol metabolism in gut metagenomes suggests that lumen microbiota of Russian ADS patients is characterized by a high potential of acetaldehyde synthesis thus contributing to the risk of colon carcinogenesis.

Furthermore, the detected dominance of the *Bifidobacterium* and *Lactobacillus* in patients with ADS and ALC points out that application of probiotic products based on the species from these two genera should be reconsidered. These bacteria have a reputation of “beneficial” agents in the human gut capable of anti-inflammatory activities, protecting against pathogens. Thus, they are being actively used for treatment of various intestinal disorders including alcohol liver disease [38]. However, our results showing enrichment of *Lactobacillus* and *Bifidobacterium* in patients suffering from alcohol dependence or liver disease suggest that these microbes could be potentially detrimental. Thus, prescription of the species to these types of patients should be approached with caution.

An additional cause for the enrichment of these two genera in guts of ADS and ALC patients could be due to involvement of these microbes in the “gut-microbiota-brain” axis. There is a frequent rate of psychological and psychiatric problems among patients suffering from alcohol abuse. One of the neuromediators playing a central role in the effect of the alcohol on the brain is serotonin [39]. Serotonin is linked to alcohol effects on the brain, and its altered levels were shown to be associated with the alcohol-related depression. It is estimated that around 80% of this neuromediator in a human organism is synthesized in the gut by enterochromaffin cells, and its synthesis has been recently shown to be regulated by the gut microbiota [40]. On the other hand, certain species of *Lactobacillus* and *Bifidobacterium* have been shown to have an effect on higher nervous functions including the behavior, with some of the species referred to as “psychobiotics” [41]. The shotgun metagenomic approach used in our study provides species-level resolution allowing one to distinguish between individual species *Bifidobacterium* and *Lactobacillus* species (the species-resolving powers of shotgun metagenomics are superior to the resolution of 16S rRNA amplicon sequencing). One of the *Bifidobacterium* species we found to be associated with liver cirrhosis is *B. dentium*. This species has been recently shown to be capable of producing large amounts of gamma-aminobutyric acid (GABA), an important neurotransmitter [42]. Based on these facts, we speculate that an increased presence of *Bifidobacterium* and *Lactobacillus* genera in the gut of alcoholics could reflect a compensatory feedback of the microbiota to altered metabolism of serotonin and other neuromediators in organisms of alcoholics. Our hypothesis could be evaluated in further experimental studies involving inoculation of germ-free animal models with species isolated from the ADS and ALC patients.

The gut dysbiosis observed in ALC and ADS patients at the taxonomic level was manifested by pronounced shifts at the level of the functional potential. Firstly, we observed significant increase of the abundance levels of the genes related to alcohol metabolism. Although this result reflects the high potential of the gut community to perform these reactions, the extent to which it manifests could be validated by measuring the expression levels using metatranscriptomic or metaproteomic techniques. Our results extend the previous observations on Japanese population showing that that feces of ADS patients display lower ethanol- and acetaldehyde-metabolizing activities than control group [29]. The difference from that study might be linked to the fact that the mentioned assay of fecal ethanol metabolism was performed under aerobic conditions, while the intestinal environment is anaerobic. The in vivo metabolic activity of gut microbiota in ADS patients should be elucidated using multi-omics experiments on larger cohorts.

Further, among functions enriched in ALC patients were glutathione metabolism (reflecting the functional adaptation of microbiota to oxidative stress associated with the inflammatory environment [43]), porphyrin metabolism and biosynthesis of siderophore group nonribosomal peptides (likely related to the iron-scavenging potential of gut pathobionts [44]), as well as ABC transporters and PTS responsible for nutrient uptake. We observed an increase in simple sugars metabolism (fructose/mannose and galactose metabolism pathways) previously reported to be enriched in subjects with inflammatory bowel disease in comparison with healthy controls [24, 45]. The observed shifts in metabolic potential of microbiota in our cohort of ADS patients were different: the list of decreased functions included phenylalanine, tyrosine, and tryptophan biosynthesis likely related to a decreased ability of the microbiota to synthesize these amino acids characteristic of auxotrophic and pathobiont species [46]. As a common feature, the functions depleted in microbiota of both ALC and ADS patients included bacterial chemotaxis and flagellar assembly. Together with a strong increase of specific gene groups related to virulence factors and metabolism of alcohol and its toxic metabolites, these results signify that gut microbiota in alcoholics might take active role in pathogenesis of associated comorbidities—to a different extent depending on the degree of the liver damage. In the future, the identified gene groups could be used as potential biomarkers in diagnostic kits for detecting gut microbiota alterations associated with pathologies linked to alcoholism and alcohol-related liver disease.

## Conclusions

This metagenomic study disentangled the impact of two main clinical factors—alcohol dependence and liver dysfunction—on the gut microbial community and its functional potential in patients suffering from alcoholic dependence. These two factors were associated with profoundly different changes in the species-level composition, suggesting that the mechanisms of microbiota involvement in the pathogenesis vary in patients with different degrees of liver damage. While specific changes associated with alcoholic dependence suggest the onset of inflammatory environment in the gut, the hallmarks of the liver cirrhosis are likely linked to the impaired bile secretion. The observation that each of these pathologies is associated with a different pool of *Bifidobacterium* and *Lactobacillus* species suggests alterations of the gut-microbiota-brain signaling in alcoholics involving these clades. Overall, our associative findings related to isolated species pave the way to understanding the altered community-level ecology within the gut community in alcohol-related diseases. Particularly, they lay the foundation for a personalized design of treatment of alcoholism in patients with different degree of liver damage. Based on patients’ microbiota composition, personalized treatment would identify the most promising pro- or/and prebiotics, as well as enable rational selection of individual donor for fecal mass transplantation (FMT)—the method whose therapeutic potential has already been demonstrated for a large number of microbiota-linked pathologies [47]. Finally, the expressed enrichment of functions related to inflammation, virulence factors, and alcohol metabolism in ALC and ADS patients suggest active contribution of gut microbiota to formation of psychiatric symptoms of alcoholic dependence, as well as to the increased risk of serious diseases associated with alcohol abuse.

## Methods

### Patients and samples

The cohort was assembled in three clinical centers (Moscow Clinical Scientific Center, Moscow; Narcology Dispensary of Republic of Tatarstan, Kazan; Saint-Petersburg Bekhterev Psychoneurological Research Institute, Saint-Petersburg) and included 99 patients with the diagnosis “alcohol dependence syndrome” (F10.(1-3) according to ICD-10) or “alcoholic liver cirrhosis” (K70.3(0-1) according to ICD-10) with age of 20 to 60 years old.

For both groups, the exclusion criteria included the presence of nonalcoholic liver diseases, decompensated diseases of other body systems, the use of pro- and/or prebiotics, nonsteroidal anti-inflammatory drugs, antibiotics, and proton pump inhibitors less than 1 month prior to the sample collection, abdominal surgery less than 3 months prior to the sample collection, alcoholic hepatic coma, and nonalcoholic liver diseases (including hepatitis B, C, and HIV). For the ALC cohort, the inclusion criteria were the presence of the alcoholic liver cirrhosis (all patients had the signs of portal hypertension) and alcohol abuse history; the exclusion criteria were the stool changes and bowel movement frequency. For the ADS, the inclusion criteria were the presence of alcohol dependence syndrome and the alcohol abuse history of at least 8 years. The exclusion criteria specific for the ADS cohort included any signs of severe liver dysfunction: the decrease of either thrombocytes, albumin, or prothrombin as well as the increase of INR.

Diagnostic criteria for the ALC patients were as follows: elevated liver enzymes (more than twice the UNL), AST to ALT ratio higher than 2:1, elevated GGT (gamma glutamyl transferase), decreased level of albumin and international normalized ratio (INR), and signs of portal hypertension (esophageal varices, ascites).

### Sample collection and metagenomic sequencing

Stool samples were collected from the subjects, stored and subjected to DNA extraction as described before [22]. Shotgun metagenomic libraries preparation and sequencing were performed using SOLiD 5500 platform (Life Technology, USA) with the following reagent kits: 5500 SOLiD Fragment Library Core Kit, SOLiD Fragment Library Barcoding Kit, SOLiD FlowChip Kit, SOLiD FWD SR S50 Kit, and SOLiD Run Cycle Buffer Kit. Barcoded fragment (nonpaired) read libraries were created from 5 μg of total DNA for each sample. The resulting read length was 50 bp.

### Taxonomic analysis of the metagenomes

The analysis of the raw metagenomic reads included quality preprocessing followed by the identification of taxonomic and functional composition (semiquantitative profiling of the abundance of the microbial taxa and functional gene groups, respectively). These analyses were performed as described before [22, 48], with the modification of the references. The reference sets included a nonredundant set of 353 gut microbial genomes (for the taxonomic analysis) [49] and a gut microbial gene catalogue of 9.9 mln genes (for the functional analysis) [50].

As an additional method for taxonomic profiling, we used MetaPhlAn v2.0 software based on the identification of unique clade-specific genetic sequences [21]. The read alignment step of MetaPhlAn was performed using Bowtie [51]. For the MetaFast analysis [23], the color-space SOLiD metagenomic reads of the patients and control group were subjected to human sequences filtering, error correction using SAET, and conversion to base-space format. The MetaFast was used with the default settings. The metagenomes were hierarchically clustered using the dissimilarity matrix; the outliers were defined as the metagenomes belonging to the smaller branch after cutting the clustering tree at the top level.

### Functional analysis of the metagenomes

The KEGG metabolic pathways differentially abundant between two groups of metagenomes were identified using piano R [52] package (parameters: gene set analysis using “reporter feature algorithm,” significance assessment using “gene sampling,” pathway significance threshold: adj. *p* < 0.05; only the pathways where at least half of the KO terms were differentially abundant were considered).

The reference gene groups associated with alcohol metabolism were selected from the KEGG metabolic pathways; their sequences related to prokaryotes were obtained. The reference sequences of genes encoding virulence factors were downloaded from the VFDB database (Virulence Factors Database) containing nucleotide sequences of 2585 genes [53].

For each virulence factor gene, the homologous genes were identified in the 9.9 mln gene catalogue using BLAST (similarity criterion: *e* value < 10^−5^, percent identity > 80% for > 80% of length). For each metagenome, the relative abundance of each reference gene was calculated by summing the abundance values of the similar genes from the abovementioned catalogue. The relative abundance of a gene from the catalogue was calculated as the number of the reads mapped to the gene normalized by the gene length and the total number of the reads mapped to the catalogue. Highly similar matches in the catalogue were identified for 402 of the 2585 gene sequences present in the VFDB.

The microbial genes related to the metabolism of alcohol/bile acids were selected from the catalogue basing on the original KO annotation of the catalogue. Relative abundance of each KO group was determined as the summary abundance of all genes belonging to that KO group.

### External metagenomic data

During the comparative analysis, the gut metagenomes from the healthy Russian population were used as an external control group (60 samples matched by the age and BMI to the ADS and ALC patients were selected from the original set of 96 metagenomes) [22]. The following published datasets were used for the multidimensional visualization: healthy subjects, patients with alcoholic and nonalcoholic liver cirrhosis from China (*n* = 69, 34 and 81) [16, 54]; healthy subjects from the USA (*n* = 139) [55] and Denmark (*n* = 85) [56].

### Statistical analysis

Statistical analysis of the microbial compositional data was performed in R [57]. The pairwise dissimilarity between the community structures was assessed using Bray-Curtis measure. The taxa or genes differentially abundant between the groups of the metagenomes were identified using Mann-Whitney rank test (multiple comparison adjustment using Benjamini-Hochberg (FDR), significance threshold: adjusted *p* < 0.01). For the comparative analysis, only the genera and species with the abundance of > 1 and > 0.1%, respectively, in at least one of the samples were included.

For the analysis of the impact of each clinical factor on the gut community structure, the list of the factors included gender, age, the presence of alcohol dependence, and the presence of liver cirrhosis. Firstly, the effect of each of the factors on the overall community structure was assessed using PERMANOVA with Bray-Curtis dissimilarity metric. Then the resulting factors significantly associated with the taxonomic composition were included into the multifactor analysis using MaAsLin package (https://huttenhower.sph.harvard.edu/maaslin). The relative abundance values of the taxa were treated as the dependent variables. The model scheme for each microbial genus/species can be summarized as follows:

*relative abundance of genus/species~alcoholic dependence + liver cirrhosis + gender*

For each subject, the factors “alcoholic dependence” and “liver cirrhosis” were assigned the values “yes” or “no” according to the group of the subject:

**Table:** 

Group	Alcoholic dependence	Liver cirrhosis
ADS	Yes	No
ALC	Yes	Yes
Control	No	No

Low-abundant taxa were filtered as described above (the final list of the taxa included 73 genera and 262 species). The MaAsLin method was used with the following parameters: the significance level was 0.05 and the dMinSamp parameter was used to limit the analysis to the taxa that were abundant at the level of > 0.01% in at least 10 samples.

The ranks of oral microbial species were compared as follows. Suppose *N* is the number of the species detected in at least one sample. For each sample, all *N* species were sorted in the decreasing order of abundance and assigned ranks (1—the most abundant, *N*—the least abundant; the species not detected in the sample were assigned *N* + 1). For each species, the respective vectors of microbial ranks were compared using Mann-Whitney test (significance threshold: *p* < 0.05).

## Additional files

Additional file 1: Table S1. Clinical data about the patients. (XLSX 14 kb)

Additional file 2: Table S2. Sequencing and general statistics of the metagenomes. (XLSX 14 kb)

Additional file 3: Table S3. Relative abundance of microbial genera in the metagenomes (the percentage of overall microbial abundance; obtained by mapping to the 353 reference gut microbial genomes). (XLSX 75 kb)

Additional file 4: Table S4. Relative abundance of microbial species in the metagenomes (the percentage of overall microbial abundance; obtained by mapping to the 353 reference gut microbial genomes). (XLSX 213 kb)

Additional file 5: Table S5. Relative abundance of microbial genera and viruses in the metagenomes (the percentage of overall abundance; obtained using MetaPhlAn2). (XLSX 96 kb)

Additional file 6: Table S6. Relative abundance of microbial species and viruses in the metagenomes (the percentage of overall abundance; obtained using MetaPhlAn2). (XLSX 185 kb)

Additional file 7: Figure S1. Heatmap displaying the relative abundance of the major microbial species for the gut metagenomes of ADS and ALC patients. Only the species with the abundance of >5% in at least one sample are shown. (PDF 115 kb)

Additional file 8: Table S7. Bacterial genera differentially abundant between each of the ADS and ALC groups and the control group (for the composition obtained via reference mapping). (XLSX 10 kb)

Additional file 9: Table S8. Bacterial species differentially abundant between each of the ADS and ALC groups and the control group (for the composition obtained via reference mapping). (XLSX 13 kb)

Additional file 10: Table S9. Bacterial genera differentially abundant between each of the ADS and ALC groups and the control group (for the composition obtained via MetaPhlAn2). (XLSX 9 kb)

Additional file 11: Table S10. Bacterial species differentially abundant between each of the ADS and ALC groups and the control group (for the composition obtained via MetaPhlAn2). (XLSX 10 kb)

Additional file 12: Figure S2. Pairwise dissimilarity of the gut metagenomes of the ADS, ALC and control groups obtained using MetaFast algorithm (Ward’s linkage, Bray-Curtis metric). (PDF 228 kb)

Additional file 13: Table S11. Associations between the levels of gut microbial genera and clinical factors. The table contains the coefficients of linear model obtained by applying MaAsLin method to the reference-mapping based taxonomic composition vectors (adjusted p-value < 0.05). Positive values denote direct association between the factor and the relative abundance of the respective taxon, while negative values denote reverse association. Each empty cell denotes that no significant association was detected between the respective factor and taxon. (XLSX 8 kb)

Additional file 14: Table S12 Associations between the levels of gut microbial species and clinical factors (MaAsLin method, adjusted p-value < 0.05). (XLSX 11 kb)

Additional file 15:Table S13. Metabolic pathways differentially abundant in ALC patients in comparison with the control group. (XLSX 8 kb)

Additional file 16: Table S14. Metabolic pathways differentially abundant in ADS patients in comparison with the control group. (XLSX 8 kb)

Additional file 17: Table S15. Gene groups related to alcohol metabolism differentially abundant in the metagenomes of ALC and ADS patients in comparison with the control group. The table contains the mean and the standard deviation of the relative abundance of KEGG Orthology Groups. (XLSX 9 kb)

Additional file 18: Table S16. Virulence factors genes with increased abundance in ALC patients in comparison with the control group. (XLSX 12 kb)

## Abbreviations

ABC: ATP-binding cassette
ADS: Alcohol dependence syndrome
ALC: Alcoholic liver cirrhosis
ALD: Alcoholic liver disease
BMI: Body mass index
FDR: False discovery rate
FMT: Fecal mass transplantation
INR: International normalized ratio
KEGG: Kyoto encyclopedia of genes and genomes
KO: KEGG orthology
MDS: Multidimensional scaling
NAFLD: Nonalcoholic fatty liver disease
NASH: Nonalcoholic steatohepatitis
PERMANOVA: Permutational analysis of variance
PTS: Phosphotransferase system
qPCR: Quantitative polymerase chain reaction
ROS: Reactive oxygen species
VFDB: Virulence factors database

## References

[1] Zaridze D, Lewington S, Boroda A, Scélo G, Karpov R, Lazarev A, et al. Alcohol and mortality in Russia: prospective observational study of 151,000 adults. The Lancet. 2014;383(9927):1465–1473. 10.1016/S0140-6736(13)62247-3 PMC4007591 24486187

[2] Sonnenburg JL, Bäckhed F (2016). Diet–microbiota interactions as moderators of human metabolism. Nature.

[3] Queipo-Ortuno MI, Boto-Ordonez M, Murri M, Gomez-Zumaquero JM, Clemente-Postigo M, Estruch R (2012). Influence of red wine polyphenols and ethanol on the gut microbiota ecology and biochemical biomarkers. Am J Clin Nutr Am Soc Nutr.

[4] Gorky J, Schwaber J (2016). The role of the gut–brain axis in alcohol use disorders. Prog Neuro-Psychopharmacol Biol Psychiatry.

[5] Leclercq S, Matamoros S, Cani PD, Neyrinck AM, Jamar F, Stärkel P, et al. Intestinal permeability, gut-bacterial dysbiosis, and behavioral markers of alcohol-dependence severity. Proc Natl Acad Sci U S A National Academy of Sciences; 2014;111:E4485-93. 10.1073/pnas.1415174111 PMC4210345 25288760

[6] Bull-Otterson L, Feng W, Kirpich I, Wang Y, Qin X, Liu Y, et al. Metagenomic analyses of alcohol induced pathogenic alterations in the intestinal microbiome and the effect of Lactobacillus rhamnosus GG treatment. Heimesaat MM, editor. PLoS One. Public Library of Science; 2013;8:e53028. 10.1371/journal.pone.0053028 PMC3541399 23326376

[7] Bajaj JS, Heuman DM, Hylemon PB, Sanyal AJ, White MB, Monteith P (2014). Altered profile of human gut microbiome is associated with cirrhosis and its complications. J Hepatol.

[8] Mutlu EA, Gillevet PM, Rangwala H, Sikaroodi M, Naqvi A, Engen PA, et al. Colonic microbiome is altered in alcoholism. Am. J. Physiol. - Gastrointest. Liver Physiol. 2012;302:G966-G978. 10.1152/ajpgi.00380.2011 PMC3362077 22241860

[9] Llopis M, Cassard AM, Wrzosek L, Boschat L, Bruneau A, Ferrere G (2016). Intestinal microbiota contributes to individual susceptibility to alcoholic liver disease. Gut.

[10] Gramenzi A, Caputo F, Biselli M, Kuria F, Loggi E, Andreone P, et al. Review article: Alcoholic liver disease - Pathophysiological aspects and risk factors. Aliment. Pharmacol. Ther. Blackwell Publishing Ltd; 2006. p. 1151–61. 10.1111/j.1365-2036.2006.03110.x 17014574

[11] Quigley EMM, Stanton C, Murphy EF (2013). The gut microbiota and the liver. Pathophysiological and clinical implications. J Hepatol.

[12] Szabo G, Bala S, Petrasek J, Gattu A (2010). Gut-liver axis and sensing microbes. Dig Dis.

[13] Abu-Shanab A, Quigley EMM (2010). The role of the gut microbiota in nonalcoholic fatty liver disease. Nat Rev Gastroenterol Hepatol.

[14] Ridlon JM, Kang D-J, Hylemon PB, Bajaj JS. Gut microbiota, cirrhosis, and alcohol regulate bile acid metabolism in the gut. Dig. Dis. NIH Public Access; 2015;33:338–345. 10.1159/000371678 PMC4470395 26045267

[15] Chen Y, Yang F, Lu H, Wang B, Chen Y, Lei D (2011). Characterization of fecal microbial communities in patients with liver cirrhosis. Hepatology.

[16] Qin N, Yang F, Li A, Prifti E, Chen Y, Shao L, et al. Alterations of the human gut microbiome in liver cirrhosis. Nature. Nature Publishing Group; 2014; 10.1038/nature13568 25079328

[17] Tuomisto S, Pessi T, Collin P, Vuento R, Aittoniemi J, Karhunen PJ (2014). Changes in gut bacterial populations and their translocation into liver and ascites in alcoholic liver cirrhotics. BMC Gastroenterol BioMed Central.

[18] Dubinkina VB, Tyakht A V., Ilina EN, Ischenko DS, Kovarsky BA, Yarygin KS, et al. Metagenomic analysis of taxonomic and functional changes in gut microbiota of patients with the alcohol dependence syndrome. Biochem. Suppl. Ser. B Biomed. Chem. Pleiades Publishing; 2016;10:184–190. 10.18097/PBMC20156106742 26716747

[19] Shalikiani NV, Bakulin IG, Dubinkina VB, Ishchenko DS, Alexeev DG, Tyakht AV (2015). Specific features of the enteric microbiota composition in patients with alcoholic liver cirrhosis. Ter Arkh.

[20] Tyakht A V, Dubinkina VB, Odintsova VY, Yarygin KS, Kovarsky BA, Pavlenko A V, et al. Data on gut metagenomes of the patients with alcoholic dependence syndrome and alcoholic liver cirrhosis. Data Br. Elsevier; 2017;11:98–102. 10.1016/j.dib.2017.01.008 PMC5257029 28138508

[21] Tyakht A V, Kostryukova ES, Popenko AS, Belenikin MS, Pavlenko A V, Larin AK, et al. Human gut microbiota community structures in urban and rural populations in Russia. Nat. Commun. Nature Publishing Group; 2013;4:2469. 10.1038/ncomms3469 PMC3778515 24036685

[22] Ulyantsev VI, Kazakov SV, Dubinkina VB, Tyakht AV, Alexeev DG (2016). MetaFast: fast reference-free graph-based comparison of shotgun metagenomic data. Bioinformatics.

[23] Morgan XC, Tickle TL, Sokol H, Gevers D, Devaney KL, Ward DV (2012). Dysfunction of the intestinal microbiome in inflammatory bowel disease and treatment. Genome Biol.

[24] Tsuruya A, Kuwahara A, Saito Y, Yamaguchi H, Tenma N, Inai M (2016). Major anaerobic bacteria responsible for the production of carcinogenic acetaldehyde from ethanol in the colon and rectum. Alcohol Alcohol.

[25] Wahlström A, Sayin SI, Marschall H-U, Bäckhed F (2016). Intestinal crosstalk between bile acids and microbiota and its impact on host metabolism. Cell Metab.

[26] Fukui H (2015). Gut-liver axis in liver cirrhosis: how to manage leaky gut and endotoxemia. World J Hepatol.

[27] Bajaj JS, Betrapally NS, Gillevet PM (2015). Decompensated cirrhosis and microbiome interpretation. Nature.

[28] Tsuruya A, Kuwahara A, Saito Y, Yamaguchi H, Tsubo T, Suga S, et al. Ecophysiological consequences of alcoholism on human gut microbiota: implications for ethanol-related pathogenesis of colon cancer. Sci. Rep. Nature Publishing Group; 2016;6:27923. 10.1038/srep27923 PMC4904738 27295340

[29] Tilg H, Mathurin P. Altered intestinal microbiota as a major driving force in alcoholic steatohepatitis. Gut. BMJ Publishing Group Ltd and British Society of Gastroenterology; 2016;65:728–9. 10.1136/gutjnl-2015-311014 26786686

[30] Wang W, Chen L, Zhou R, Wang X, Song L, Huang S, et al. Increased proportions of Bifidobacterium and the Lactobacillus group and loss of butyrate-producing bacteria in inflammatory bowel disease. J. Clin. Microbiol. American Society for Microbiology; 2014;52:398–406. 10.1128/JCM.01500-13 PMC3911339 24478468

[31] Taur Y, Xavier JB, Lipuma L, Ubeda C, Goldberg J, Gobourne A (2012). Intestinal domination and the risk of bacteremia in patients undergoing allogeneic hematopoietic stem cell transplantation. Clin Infect Dis.

[32] Veiga P, Pons N, Agrawal A, Oozeer R, Guyonnet D, Faurie J (2014). Changes of the human gut microbiome induced by a fermented milk product ´. Sci Rep.

[33] Nosova T, Jokelainen K, Kaihovaara P, Heine R, Jousimies-Somer H, Salaspuro M. Characteristics of aldehyde dehydrogenases of certain aerobic bacteria representing human colonic flora. Alcohol Alcohol. 1998;33:273–80. 10.1093/oxfordjournals.alcalc.a008391 9632053

[34] Peng Y, Shi H, Qi X, Xiao C, Zhong H, Ma RZ (2010). The ADH1B Arg47His polymorphism in East Asian populations and expansion of rice domestication in history. BMC Evol Biol.

[35] Leon DA, Saburova L, Tomkins S, Andreev E, Kiryanov N, McKee M (2007). Hazardous alcohol drinking and premature mortality in Russia: a population based case-control study. Lancet.

[36] McKee M, Suzcs S, Sárváry A, Adany R, Kiryanov N, Saburova L (2005). The composition of surrogate alcohols consumed in Russia. Alcohol Clin Exp Res.

[37] Vassallo G, Mirijello A, Ferrulli A, Antonelli M, Landolfi R, Gasbarrini A (2015). Review article: alcohol and gut microbiota—the possible role of gut microbiota modulation in the treatment of alcoholic liver disease. Aliment Pharmacol Ther.

[38] Lovinger DM (1997). Serotonin’s role in alcohol’s effects on the brain. Alcohol Health Res World.

[39] Yano JM, Yu K, Donaldson GP, Shastri GG, Ann P, Ma L (2015). Indigenous bacteria from the gut microbiota regulate host serotonin biosynthesis. Cell.

[40] Dinan TG, Stanton C, Cryan JF (2013). Psychobiotics: a novel class of psychotropic. Biol Psychiatry.

[41] Pokusaeva K, Johnson C, Luk B, Uribe G, Fu Y, Oezguen N (2017). GABA-producing *Bifidobacterium dentium* modulates visceral sensitivity in the intestine. Neurogastroenterol Motil.

[42] Masip L, Veeravalli K, Georgiou G (2006). The many faces of glutathione in bacteria. Antioxid Redox Signal.

[43] Kortman GAM, Raffatellu M, Swinkels DW, Tjalsma H (2014). Nutritional iron turned inside out: intestinal stress from a gut microbial perspective. FEMS Microbiol Rev.

[44] Lewis JD, Chen EZ, Baldassano RN, Otley AR, Griffiths AM, Lee D (2015). Inflammation, antibiotics, and diet as environmental stressors of the gut microbiome in pediatric Crohn’s disease. Cell Host Microbe.

[45] Kostic AD, Xavier RJ, Gevers D (2014). The microbiome in inflammatory bowel disease: current status and the future ahead. Gastroenterology.

[46] Rossen NG, MacDonald JK, de Vries EM, D’Haens GR, de Vos WM, Zoetendal EG, et al. Fecal microbiota transplantation as novel therapy in gastroenterology: a systematic review. World J. Gastroenterol. Baishideng Publishing Group Inc; 2015;21:5359–71. 10.3748/wjg.v21.i17.5359 PMC4419078 25954111

[47] Yarygin K, Tyakht A, Larin A, Kostryukova E, Kolchenko S, Bitner V (2017). Abundance profiling of specific gene groups using precomputed gut metagenomes yields novel biological hypotheses. PLoS One.

[48] Dubinkina VB, Ischenko DS, Ulyantsev VI, Tyakht AV, Alexeev DG (2016). Assessment of k-mer spectrum applicability for metagenomic dissimilarity analysis. BMC Bioinf.

[49] Li J, Jia H, Cai X, Zhong H, Feng Q, Sunagawa S, et al. An integrated catalog of reference genes in the human gut microbiome. Nat. Biotechnol. 2014;32(8):834-841. 10.1038/nbt.2942 24997786

[50] Langmead B, Trapnell C, Pop M, Salzberg SL (2009). Ultrafast and memory-efficient alignment of short DNA sequences to the human genome. Genome Biol.

[51] Väremo L, Nielsen J, Nookaew I. Enriching the gene set analysis of genome-wide data by incorporating directionality of gene expression and combining statistical hypotheses and methods. Nucleic Acids Res. 2013;41:4378–91. 10.1093/nar/gkt111 PMC3632109 23444143

[52] Chen L, Xiong Z, Sun L, Yang J, Jin Q (2012). VFDB 2012 update: toward the genetic diversity and molecular evolution of bacterial virulence factors. Nucleic Acids Res.

[53] Qin J, Li Y, Cai Z, Li S, Zhu J, Zhang F, et al. A metagenome-wide association study of gut microbiota in type 2 diabetes. Nature. Nature Publishing Group; 2012;490:55–60. 10.1038/nature11450 23023125

[54] The Human Microbiome Project Consortium. Structure, function and diversity of the healthy human microbiome. Nature. Nature Publishing Group; 2012;486:207–14. 10.1038/nature11234 PMC3564958 22699609

[55] Qin J, Li R, Raes J, Arumugam M, Burgdorf KS, Manichanh C, et al. A human gut microbial gene catalogue established by metagenomic sequencing. Nature. Macmillan Publishers Limited. All rights reserved; 2010;464:59–65. 10.1038/nature08821 PMC3779803 20203603

[56] R Development Core Team. R: A Language and environment for statistical computing. Vienna Austria R Found. Stat. Comput. 2008. p. 2673.

[57] Tyakht A V, Kostryukova ES, Popenko AS, Belenikin MS, Pavlenko A V, Larin AK, et al. Human gut microbiota community structures in urban and rural populations in Russia. Nat. Commun. Nature Publishing Group; 2013;4. 10.1038/ncomms3469 PMC3778515 24036685

[58] Truong DT, Franzosa EA, Tickle TL, Scholz M, Weingart G, Pasolli E (2015). MetaPhlAn2 for enhanced metagenomic taxonomic profiling. Nat Methods.

