# Supplementary figures and images for: Links of gut microbiota composition with alcohol dependence syndrome and alcoholic liver disease
Source: Microbiome. 2017 Oct 17;5:141. doi: 10.1186/s40168-017-0359-2 (PMC5645934; doi:10.1186/s40168-017-0359-2)

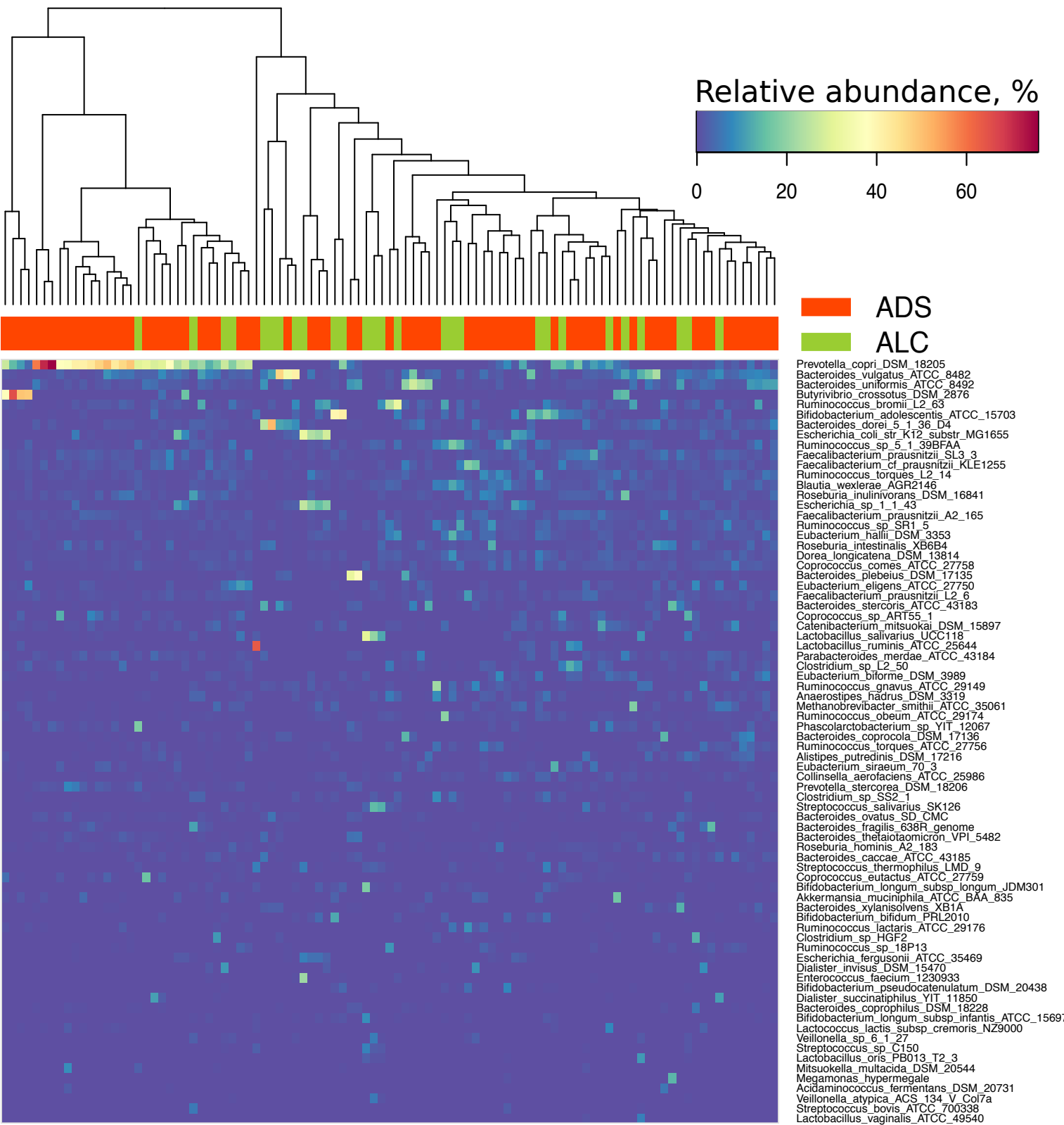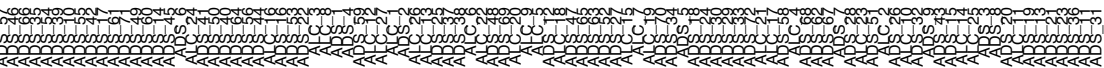

Supplement: Supplementary file 7 — Heatmap displaying the relative abundance of the major microbial species for the gut metagenomes of ADS and ALC patients. Only the species with the abundance of >5% in at least one sample are shown. (PDF 115 kb) [file 40168_2017_359_MOESM7_ESM.pdf]

# Color Key

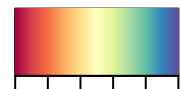

0 0.4 0.8

Value

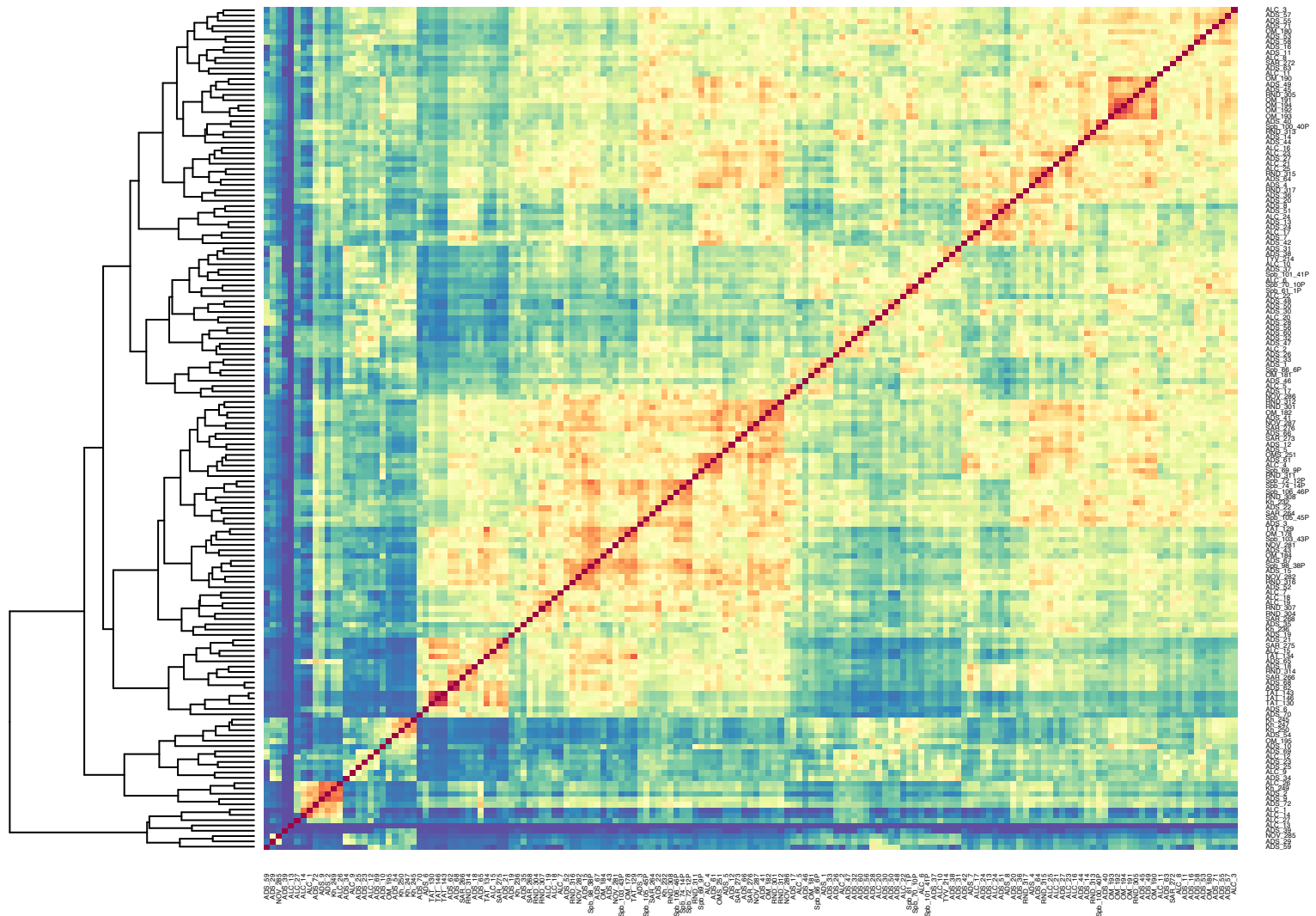

Supplement: Supplementary file 12 — Pairwise dissimilarity of the gut metagenomes of the ADS, ALC and control groups obtained using MetaFast algorithm (Ward’s linkage, Bray-Curtis metric). (PDF 228 kb) [file 40168_2017_359_MOESM12_ESM.pdf]
